# Comparison of simultaneous bilateral CI surgery vs. sequential CI surgery regarding operative time, perioperative morbidity, and anesthesia risk in children

**DOI:** 10.3389/fneur.2025.1660108

**Published:** 2025-10-09

**Authors:** Leonie Fries, Friederike Everad, Rainer Linus Beck, Antje Aschendorff, Susan Arndt, Manuel Christoph Ketterer

**Affiliations:** Department of Otorhinolaryngology, Medical Center—University of Freiburg, Faculty of Medicine, University of Freiburg, Freiburg, Germany

**Keywords:** bilateral cochlear implant, bilateral CI, sequential cochlear implantation, simultaneous cochlear implantation, cochlear implantation in children

## Abstract

**Objectives:**

In recent years, simultaneous bilateral cochlear implantation (CI) has become the preferred procedure for children with bilateral deafness. The aim of this study is to compare simultaneous bilateral implantation with sequential bilateral CI concerning duration of surgery and anesthesia, perioperative morbidity, mortality, and anesthesia risk.

**Methods:**

A retrospective data analysis was conducted on 132 children who were implanted between 2019 and 2024 and aged under 10 years at the time of either simultaneous or second CI. The age at implantation, duration of surgery and anesthesia, anesthesia risk, perioperative morbidity and mortality were compared between the simultaneous and the sequential implantation cohorts.

**Results:**

Simultaneous bilateral CI demonstrated a significant reduced duration of anesthesia in total for both sides (simultaneous CI: 221.7 ± 41.4 min; sequential CI: 262.3 ± 37.11 min, *p* < 0.0001) and a significant shorter duration of surgery with a mean of 108.6 min (±38.2 min) for both sides in simultaneous CI and 132.7 min (±36.85 min) for sequential CI (*p* < 0.0001). However, perioperative morbidity and mortality as well as anesthesia risk showed no significant differences. The cumulative duration of hospitalization was significantly longer for sequential CI (simultaneous CI: 5 ± 0.76 days; sequential CI: 10 ± 0.85 days).

**Conclusion:**

The study underlines the benefits of simultaneous bilateral CI, regarded as the gold standard, particularly concerning reduced duration of surgery and anesthesia time. In an era of healthcare cost efficiency, simultaneous CI also enables shorter hospital stays. However, the absence of significant differences in perioperative morbidity, mortality, and anesthesia risks must be considered. This makes sequential CI a viable treatment alternative and allows for an individualized treatment approach that accounts for existing comorbidities and individual patient and parental factors.

## Introduction

Cochlear implantation (CI) is well established and performed for children with prelingual deafness and severe-profound sensorineural hearing loss as well as progressive hearing loss (1, 2). With a prevalence of hearing impairment in childhood and adolescence of 1–4% and a prevalence of profound hearing loss of 0.1% in Germany and 1.7 in 1,000 children born with bilateral deafness in the United States of America, this is an important issue that requires a lot of medical and financial resources (3, 4). Bilateral CI can restore binaural hearing, which then allows for better speech discrimination in silence and noise, as well as better sound localization, explained by mechanisms such as squelch effect, head shadow effect, and summation effect (5–7). In children with bilateral hearing impairment early bilateral CI is important for their speech development as it relies on their hearing ability (6, 8, 9). The first bilateral CI was performed in 1996 followed by the first bilaterally implanted child in 1998, being now a standard treatment for patients with bilateral severe to profound sensorineural hearing loss (6, 10). Several studies comparing bilateral with unilateral CI exist which indicate an additional benefit for the second CI especially in sound localization and speech development (6, 8, 11–14). While there is no doubt for bilateral CI in bilateral deaf patients, there still exist controversies in sequential versus simultaneous CI. Simultaneous CI has become the standard procedure in most countries as early implantation and shorter inter-implant intervals are considered to improve speech recognition and hearing performance (2, 15–18). Despite that, the cost-effectiveness presents another argument for simultaneous implantation (19, 20). In contrast to the existing recommendation for simultaneous CI in bilaterally deaf children, some centers still prefer to perform sequential implantation due to lower estimated perioperative morbidity. Major argument performing sequential CI is a possible total bilateral vestibular loss and the possible loss of residual hearing mostly important for non-users as well as additional anesthetic and surgical risks (8, 11).

The present study is a retrospective review of the surgical outcomes of CI over a six-year period. The aim of our study was to examine the differences between simultaneous bilateral CI and sequential bilateral CI. In particular, the two surgical modalities were contrasted for cumulative duration of surgery and anesthesia as well as the perioperative morbidity and mortality. Furthermore, we collected information on the length of hospital stay, as CI surgery in children usually is an inpatient procedure in Germany.

## Materials and methods

We performed a retrospective analysis of children with bilateral hearing loss or profound hearing impairment receiving a CI between 2019 and 2024. The study was performed in the Department of Otorhinolaryngology, Head and Neck surgery, at the Implant Center of the University Hospital Freiburg. Patients aged under 10 years at the time of either simultaneous or second CI were included regardless of the cause of hearing impairment. CI surgery was performed in our department of six different surgeons, patients implanted in another clinic or with missing implantation data were excluded. We included both patients for whom we recommended direct bilateral CI at the first presentation and patients who were initially adequately fitted with hearing aids but were meeting the indication criteria for CI later at a second or third presentation due to progressive hearing loss. Records were retrospectively analyzed for age at implantation, duration of surgery and anesthesia, anesthesia risk, perioperative morbidity and mortality as well as pre-existing illnesses and syndromes. These were compared between the simultaneous and the sequential implantation cohorts. The decision for simultaneous or sequential implantation was made either primarily by the parents after discussion and counseling with the surgeons in the case of CI indication for both ears or by the surgeons who evaluated the indication criteria based on residual hearing.

The statistical analysis was performed using Prism 7-software (GraphPad Software, Inc., La Jolla, CA, United States). We used the nonparametric Mann–Whitney test for statistical analysis. For calculating the results significance level of *p* < 0.05 was used in all statistical analyses. Approval was obtained from the hospital ethics committee in accordance with the Declaration of Helsinki (Washington, 2002) (ethics committee approval number: 406/19 amendment number: 230282).

## Results

### Study cohort

In total 132 children implanted with CI between 2019 and 2024 were included in this study. Among the 132 patients, 92 patients were implanted simultaneously and 40 patients were implanted sequentially. The mean age at implantation in the simultaneous cohort was 20.09 months, ranging from 6 months to 90 months, while the mean age at implantation for the first CI in the sequential cohort was 39 months, ranging from 9 months to 110 months, and the second CI was 63.33 months, ranging from 23 months to 146 months (see Table 1). In total 30.3% (40/132) patients were 12 months old or younger. 35 out of 92 children (38.0%) in the simultaneous cohort were 12 months old or younger. In the sequential cohort, however, only five out of 40 children (12.5%) were 12 months old or younger. 64 children were female. For sequential CI the mean inter-implant interval was 26.16 months (± 20.6). Mean time of cochlear implantation was 23.7 months (±12.95) after diagnosis of deafness for the sequential cohort and 16.7 months (± 9.03) for the simultaneous cohort.

**Table 1:** Descriptive study cohort details comparing simultaneous CI and sequential CI.

	Simultaneous CI	Sequential CI
Patients (*n*)	92	40
Age (months) [mean, (IQR; min–max)]	20.09 (66; 6–90)	First surgery: 39.0 (40,25; 9–110) Second surgery: 63.33 (37; 23–146)
Inter-implant interval (months) (mean, SD)	0	26.16 (20.6)

IQR, interquartile range; SD, standard deviation; min, minimum; max, maximum.

### Operating times

**Figure 1:**
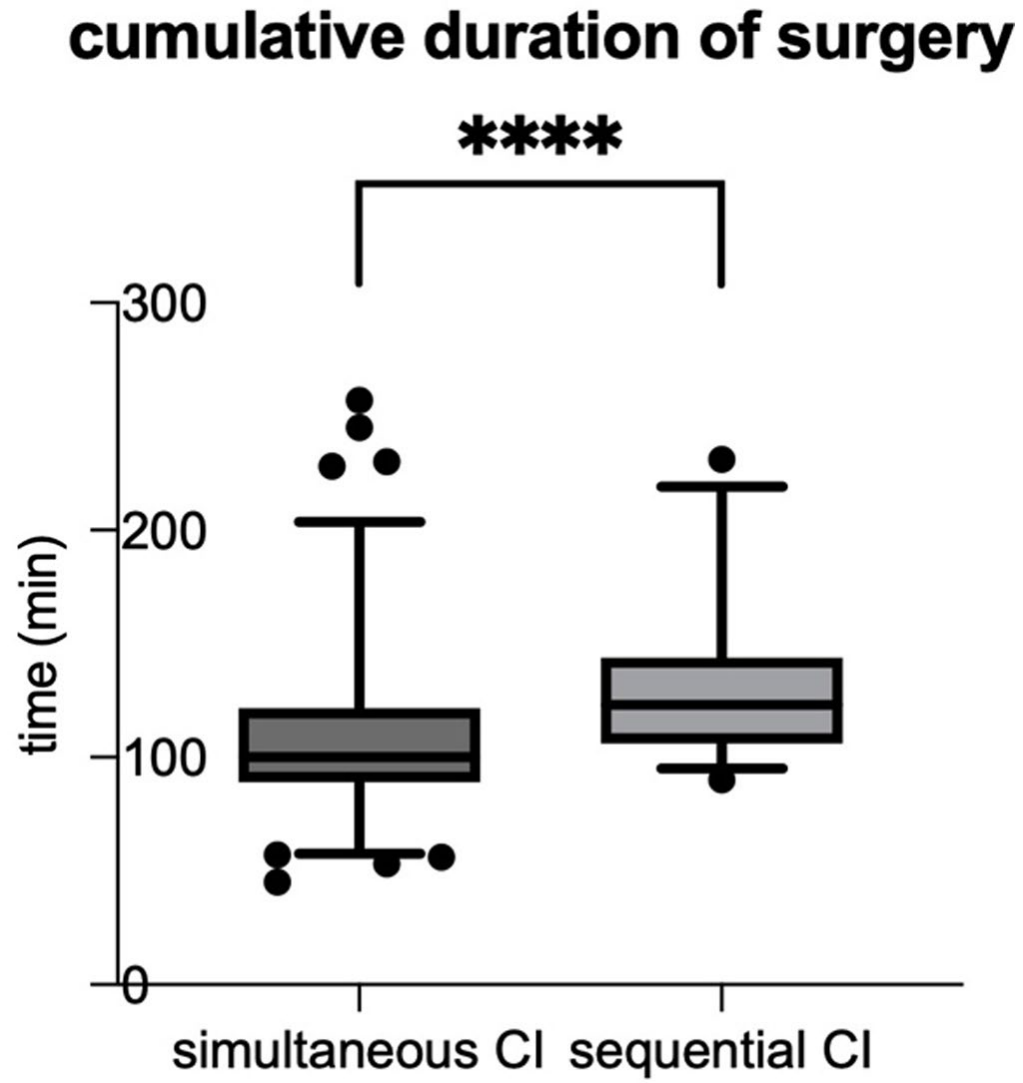
The cumulative duration of surgery (time from incision to suture for each side) was significantly shorter for simultaneous CI compared to sequential CI (simultaneous CI: 108.6 ± 38.20 min; sequential CI: 132.7 ± 36.85 min; ^****^p < 0.0001).

The cumulative duration of surgery was defined as the time from incision to suture, with the times for each side being added. The mean cumulative duration of surgery (from incision to suture for each side) in the cumulative cohort was significantly shorter than in the sequential cohort (*p* < 0.0001) (see Table 2; Figure 1). When comparing the surgical duration of the first implanted ears between the two cohorts the effects remain the same with shorter duration of surgery for simultaneous CI, which can also be seen for the second ears (see Table 2).

**Table 2:** Duration of surgery and anesthesia compared between simultaneous and sequential cohort.

	Simultaneous CI	Sequential CI	*p*
Cumulative duration of surgery in min. (incision to suture for each side) (mean, SD)	108.6 (38.2)	132.7 (36.85)	<0.0001
Duration of surgery (first ear) in min. (mean, SD)	55.69 (18.15)	72.75 (32.01)	<0.0002
Duration of surgery (second ear) in min. (mean, SD)	57.94 (22.92)	59.38 (11.51)	<0.0147
Cumulative duration of anesthesia in min. (mean, SD)	221.7 (41.4)	262.3 (37.11)	<0.0001

Min, minutes; SD, standard deviation.

**Figure 2:**
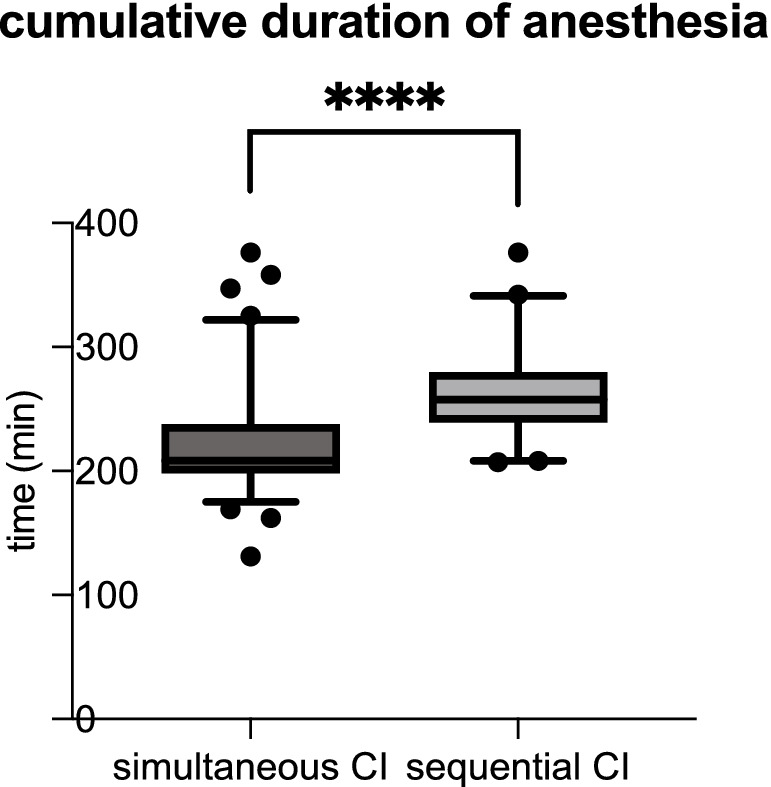
The cumulative duration of anesthesia was significantly shorter for simultaneous CI than sequential CI (simultaneous CI: 221.7 ± 41.40 min; sequential CI: 262.3 ± 37.11 min; ^****^p < 0.0001).

Whereas the mean duration of anesthesia is 262.3 min for sequential CI, it is significantly shorter for simultaneous CI with a mean anesthesia time of 221.7 min (see Table 2; Figure 2).

### Anesthesia risk, perioperative morbidity and mortality, hospital stay

**Figure 3:**
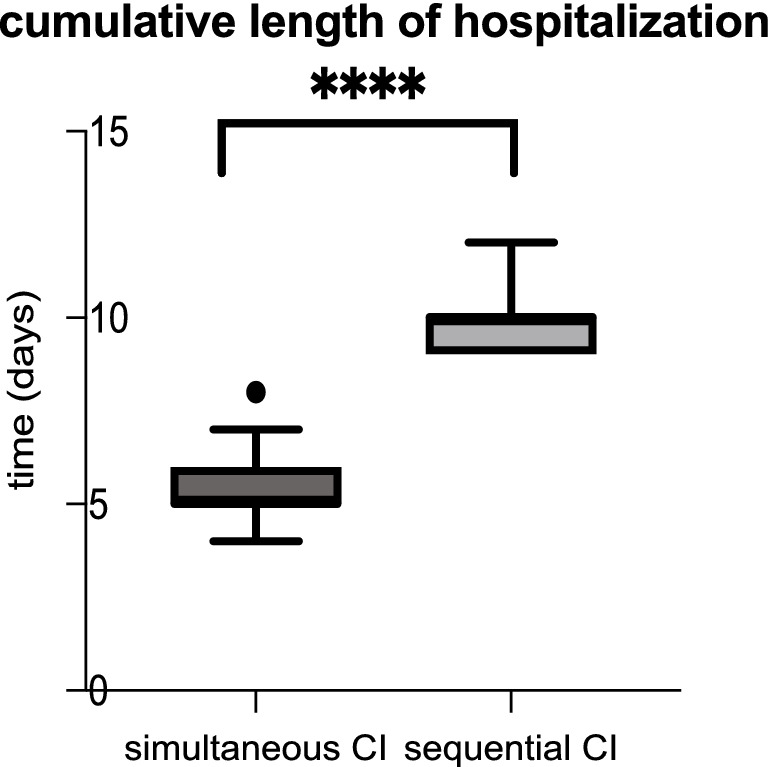
Cumulative hospital stay was significantly longer for sequential CI (*p* < 0.0001).

Comparing the anesthesiological complications between the two cohorts, there was no significant difference. Roughly 10% of patients with simultaneous CI as well as 10% of sequential CI showed minor events during anesthesia as prolonged time to extubation, bradycardia, bronchial reaction and difficult intubation (see Table 3). As shown in Table 3, perioperative complications such as hematoma, wound infection, fever, vertigo, pain exacerbation and cerebrospinal fluid (CSF) leak were rare in both, sequential and simultaneous CI, but more frequent with sequential CI. Whereas three patients with simultaneous CI had wound infection, two had fever and one each had postoperative CSF leak and hematoma, two patients with sequential CI experienced vertigo, one had pain exacerbation, and one had fever (see Table 4). The patient with a postoperative CSF leak underwent revision surgery involving the proper sealing of the cochleostomy 7 days after the initial CI. The 11-month-old patient was diagnosed with Johanson-Blizzard syndrome, which is characterized by cochlear malformation and hypoplasia. Of the two patients who underwent sequential CI, one patient who was 47 months old, experienced vertigo the day after surgery, while the other experienced vertigo 7 days postoperatively, requiring inpatient treatment. No fatal complications occurred in any of the patients who received either sequential or simultaneous CI (Table 3). Comparing the perioperative complications between the young children (12 months or younger) and the elder children with a least 13 months of age there was no statistical difference with 4/132 being younger or 12 months old and 7/132 being older or 13 months old. There was no statistical difference in perioperative complications between young children (12 months or younger) and older children (at least 13 months old). 4 out of 40 (10.0%) of the younger children and 7 out of 92 (7.6%) of the older children presented with minor postoperative complications. In terms of anesthesiological risk, complications occurred in 10.8% (10/92) of older children (>12 months), while only 7.5% (3/40) of younger children had minor complications. The mean hospital stay was 5.24 days for simultaneous CI and 10.03 days for sequential CI, with the latter being calculated as the sum of the durations of both surgeries (see Figure 3). Hospitalization was significantly longer for sequential CI with twice the length of hospital stay (see Table 3; Figure 3).

**Table 3:** Perioperative mortality as well as anesthesia risk and anesthesiological complications showed no significant differences.

	Simultaneous CI	Sequential CI	*p*
Anesthesiological complications [%, (*n*)]^*^	9.8% (9/92)	10% (4/40)	ns
Perioperative minor complications [%, (*n*)]^**^	7.6% (7/92)	10% (4/40)	ns
Perioperative mortality [%, (*n*)]	0% (0/92)	0% (0/40)	ns
Length of hospitalization (days, SD)	5.24 (0.76)	10.03 (0.84)	ns

Perioperative minor complications were more frequent with sequential CI without being statistically significant. ^*^Prolonged time to extubation, bradycardia, bronchial reaction, difficult intubation; ^**^minor-complications: hematoma, wound infection, fever, vertigo, pain exacerbation, liquor leak; ns, not significant; SD, standard deviation.

**Table 4:** Perioperative complications were rare in both groups, but more frequent with sequential (4/40, 10%) than simultaneous (7/92, 7.6%) CI.

Perioperative minor complications	Simultaneous CI	Sequential CI
Wound infection [%, (*n*)]	3.3% (3/92)	0% (0/40)
Pain exacerbation [%, (*n*)]	0% (0/92)	2.5% (1/40)
Fever [%, (*n*)]	2.2% (2/92)	2.5% (1/40)
Hematoma [%, (*n*)]	1.1% (1/92)	0% (0/40)
Vertigo [%, (*n*)]	0% (0/92)	5% (2/40)
CSF leak [%, (*n*)]	1.1% (1/92)	0% (0/40)

### Etiology of hearing loss, causes for sequential CI and comorbidities

**Figure 4:**
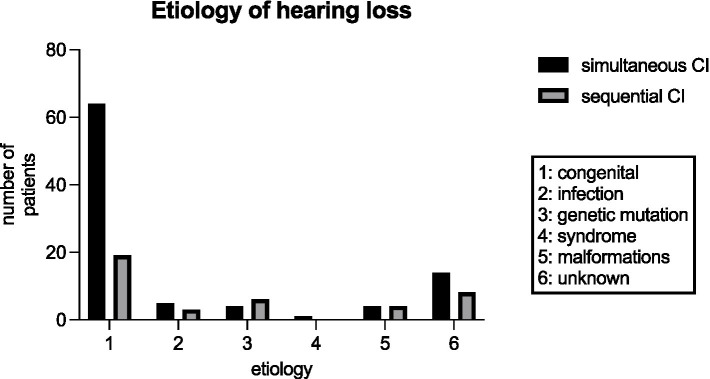
Etiology of hearing loss for patients with simultaneous CI and sequential CI in our study cohort.

**Figure 5:**
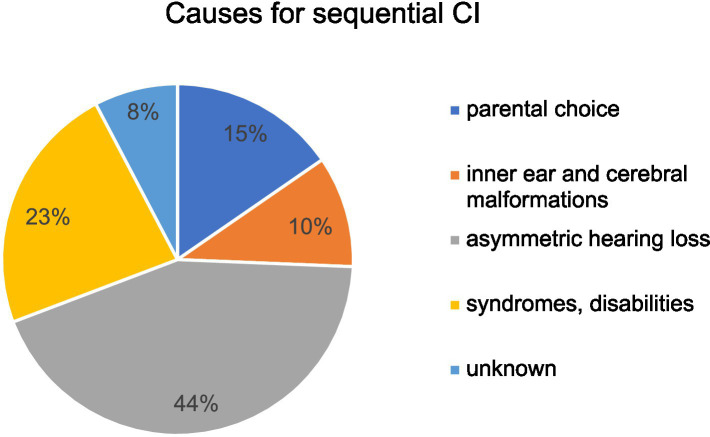
Causes for performing sequential CI in our study cohort.

Figure 4 shows the etiologies of hearing loss for simultaneous versus sequential CI. While 69.6% (64/92) of patients in the cohort of simultaneous CI presented with congenital hearing loss only 47.5% (19/40) of patients with congenital hearing loss underwent sequential CI. Infection was a rare cause of hearing loss in both groups but was more common in patients with sequential CI (see Figure 4). A GJB2 gene mutation was found in 4.3% (4/92) of patients with simultaneous CI, whereas 15% (6/40) of patients with sequential CI had a genetic disorder such as mutations in GJB2 gene, DFNBA1 gene and LOXHD1 gene. Four patients in each group had an inner ear malformation (see Figure 4). In our study group, the most common reason for choosing a sequential CI was asymmetric hearing loss (AHL), with progressive hearing loss on the not primarily implanted side occurring in 42.5% of cases (17 out of 40). In 15%, the sequential CI was performed due to parental choice. Other reasons for the decision to proceed sequential CI were inner ear and cerebral malformations in 4 cases, syndromes and other disabilities that might compromise the benefit of CI in 9 cases and 3 cases of unknown cause according to the retrospective analysis of the medical records (see Figure 5). Comorbidities were known in 15 of 92 patients in the simultaneous CI cohort and 11 of 40 patients in the sequential CI cohort. Three patients with simultaneous CI and four patients with sequential CI had cerebral and facial anomalies, such as cysts in the pituitary or pineal gland, spinocerebellar ataxia, unilateral cerebral palsy, orofacial cleft lip and previous intracerebral hemorrhage. Motor development delay and lung disorders were also present in one patient with each disease in both groups. Two patients with sequential CI and one with simultaneous CI had hematological diseases. Congenital cardiovascular defects were reported in two children with simultaneous CI and one child with sequential CI. While three patients in the simultaneous cohort had a syndromal disorder (one with Zellweger syndrome, one with Johanson-Blizzard syndrome, one with West syndrome), none of the patients in the sequential cohort did. In the sequential cohort one child presented with renal dysplasia and another child with atopic dermatitis. Two children in the simultaneous cohort were born prematurely.

## Discussion

The performance of CI in bilaterally deaf children or children with severe-profound sensorineural hearing loss at a young age is essential for enhancing speech recognition and production (8, 13). Bilateral implantation has proven to enhance speech performance and sound localization. This procedure may be executed in either a simultaneous or sequential manner. In our study 132 patients were included and retrospectively evaluated. Among the 92 patients being implanted simultaneously the age was younger than in the sequential cohort with a mean of approximately 20 months, and the youngest child was implanted at the age of 6 months. This phenomenon can be explained by the earlier implantation of children in the simultaneous cohort caused by the elevated prevalence of deafness in the sample. In contrast, the sequential cohort comprises a substantial number of children who present with AHL and progression of the second ear over time.

In addition, also 16 children with bilateral deafness underwent sequential implantation. Our data demonstrates an inter-implant interval of 26.16 months between surgeries. While another study describes a mean inter-implant interval of 58 months (range 3–143 months), Uecker et al. (12, 21) showed an exemplary average of 10 months between the surgeries differing from other studies with an interval of around 19 months. The increased inter-implant interval observed in the present study is partly due to the manifestation of hearing loss in the second ear over time, resulting in a longer latency to reach the established inclusion criteria. Another reason for delayed implantation of the second ear is related to the fact that families may not be aware of the need for bilateral implantation, which should be evaluated regularly during follow-up. Given the documented inferior outcomes observed in speech performance in cases involving delayed implantation of the second ear in children with bilateral profound hearing loss, it is imperative to minimize the delay in such cases (15). Concomitantly, it is necessary to consider the heightened surgical risk associated with performing surgical procedures on very young infants (22). Lately, studies have shown that CI in infants under the age of 12 months is safe with appropriate anesthesia and postoperative management in centers with advanced pediatric experience (22–24). In our study cohort, no significant differences in anesthesiological or perioperative complications were observed between younger children (12 months old or younger) and older children (over 12 months old). In addition, the youngest child in our study underwent simultaneous CI at the age of 6 months without experiencing any anesthesiological or perioperative complications. Our study shows a significant reduction of surgical duration for simultaneous CI compared to the cumulative time needed for sequential surgery. The findings of our study are consistent with previous literature (21, 25). Furthermore, the findings of this study demonstrate that the cumulative anesthesia time for simultaneous CI is shorter than for sequential CI. This is an important consideration for children with predominant illnesses. However, it should be noted that operating one ear after the other does exhibit a shorter duration of anesthesia for one ear. This approach may be considered for children with elevated anesthesiological risks, such as those with lung disorders. Comparing the anesthesiological risk there is no difference between the two surgery modalities with both cohorts showing roughly 10% minor complications such as bradycardia, bronchial reaction or prolonged extubating in our study. Regarding minor surgical complications such as fever, vertigo, pain exacerbation and hematoma also no statistical difference was demonstrated. This is concordant with the data of a previous study also comparing bilateral CI (14, 21, 26). Nevertheless, there is also evidence indicating elevated risks of perioperative complications for simultaneous CI, which can be reduced by an assumed learning curve of surgeons in the recent years (16, 27). In summary, the present study indicates that simultaneous bilateral CI in childhood does not result in a higher incidence of complications when compared with the sequential implantation approach. Consistent with Uecker et al. (21), in our data the simultaneous implantation required a significantly reduced hospitalization time. The increase in hospital stays up to twice the duration for sequential CI (a total of 10 days for sequential CI and 5 days for simultaneous CI) also compromises cost-effectiveness, resulting in higher costs for the sequential procedure. Nevertheless, the total increase in hospitalization time reflects the current treatment modalities according to the German CI guidelines. In addition to the reduced length of hospital stay, cost efficiency may be achieved through the economical use of surgical time and materials with one simultaneous surgery versus two required surgeries. Indirect costs due to parents being absent from work or the need to organize childcare for other children are also reduced with the simultaneous approach (21).

In our study cohort, only two sequentially implanted patients experienced postoperative vertigo, which is a rare risk of CI. Of the two patients who underwent sequential CI, one patient who was 47 months old, experienced vertigo the day after surgery, while the other experienced vertigo 7 days postoperatively, requiring inpatient treatment. This is one of the main arguments of the critics of simultaneous surgery and must be taken into account when deciding on the surgical modality (8, 11).

The aforementioned arguments illustrate the advantages of concurrent CI, thereby prompting the inquiry into the continued necessity of sequential CI. As previously stated, sequential CI remains a viable option for patients with predominant illnesses, such as those requiring a shorter duration of anesthesia or blood-clotting disorders. Additionally, it may be considered for patients with special malformations or syndromes for whom the benefit of a CI is uncertain. Especially in latter, this is a valuable option for parents who are still skeptical about undergoing surgery. In summary, the two procedures should be thoroughly discussed interdisciplinary by the healthcare team and the families. Despite the study’s notable strength in including the largest number of patients to date who are comparing the simultaneous and sequential CI, the number of cases is insufficient for conducting further statistical evaluation or subgroup analysis. This limitation is particularly problematic when it comes to considering the influence of the patient’s age or prevailing risk factors. A further limitation is posed by the retrospective design, which precludes comprehension of the decision-making process undertaken by parents and the healthcare team. This may result in the occurrence of selection bias. Moreover, the duration of surgery and anesthesia vary, a discrepancy that can be partially attributed to the varying experience of the participating surgeons and anesthesiologists.

Although vertigo is rare in children with CI, it has been mentioned as a critical factor in the discussion of simultaneous implantation in several studies (8, 11). In our study, only two children experienced postoperative vertigo, both of which resolved completely. However, and this must be considered a limitation of this study, adequate functional diagnostic tests were not available due to the retrospective nature of this study. Therefore, vestibular dysfunction in CI children should be prospectively investigated in future studies.

## Conclusion

Bilateral simultaneous CI is a sufficient and safe surgical procedure for bilaterally deaf children or children with bilateral severe-profound sensorineural hearing loss. The outlining benefits are the shortened duration of surgery and anesthesia as well as the shorter hospitalization resulting in lower healthcare costs. However, the absence of significant differences in perioperative morbidity, mortality, and anesthesia risks must be considered and make the sequential CI a considerable treatment alternative. Our data illustrate the advantages of simultaneous CI in case of bilateral CI indication as the preferable option, while emphasizing the necessity of a thorough preoperative evaluation. Sequential CI is still offering an individualized treatment approach that considers existing comorbidities and individual patient and parental factors.

## Data Availability

The original contributions presented in the study are included in the article/supplementary material, further inquiries can be directed to the corresponding author.
